# Specific Enriched Acinetobacter in *Camellia* Weevil Gut Facilitate the Degradation of Tea Saponin: Inferred from Bacterial Genomic and Transcriptomic Analyses

**DOI:** 10.1128/spectrum.02272-22

**Published:** 2022-11-22

**Authors:** Zikun Li, Suya Huang, Xinghua He, Haijie Ma, Xudong Zhou, Haiping Lin, Shouke Zhang

**Affiliations:** a State Key Laboratory of Subtropical Silviculture, Zhejiang A & F University, Hangzhou, People’s Republic of China; b College of Forestry and Biotechnology, Zhejiang A & F University, Hangzhou, People’s Republic of China; c College of Horticulture Science, Zhejiang A & F University, Hangzhou, People’s Republic of China; d Zhoushan Academy of Forestry Science, Zhoushan, People’s Republic of China; University of Minnesota

**Keywords:** gut microbiome, triterpenoids, plant secondary metabolite, *Acinetobacter*, resistance, tea saponin, tolerance

## Abstract

Beneficial gut bacteria can enhance herbivorous arthropod adaptation to plant secondary compounds (PSMs), and specialist herbivores provide excellent examples of this. Tea saponin (TS) of *Camellia oleifera* is triterpenoids toxic to seed-feeding weevil pest, *Curculio chinensis* (CW). Previous studies disclosed that Acinetobacter, which was specific enriched in the CW’s gut, was involved in helping CW evade TS toxicity of *C. oleifera*. However, it is still not clear whether Acinetobacter is associated with other anti-insect compounds, and the molecular mechanism of Acinetobacter degradation of TS has not been clarified. To address these questions, we explored the relationship between host plant toxin content and Acinetobacter of CW gut bacteria. Results demonstrated that TS content significantly affected the CW gut microbiome structure and enriched bacteria functional for TS degradation. We further isolated Acinetobacter strain and conducted its genome and transcriptome analyses for bacterial characterization and investigation on its role in TS degradation. Biological tests were carried out to verify the ability of the functional bacterium within CW larvae to detoxify TS. Our results showed that TS-degrading bacteria strain (Acinetobacter sp. AS23) genome contains 47 genes relating to triterpenoids degradation. The AS23 strain improved the survival rate of CW larvae, and the steroid degradation pathway could be the key one for AS23 to degrade TS. This study provides the direct evidence that gut bacteria mediate adaptation of herbivorous insects to phytochemical resistance.

**IMPORTANCE** Microorganism is directly exposed to the plant toxin environment and play a crucial third party in herbivores gut. Although previous studies have proved the existence of gut bacteria that help CWs degrade TS, the specific core flora and its function have not been explored. In this study, we investigated the correlation between the larva gut microbiome and plant secondary metabolites. Acinetobacter genus was the target flora related to TS degradation. There were many terpenoids genes in Acinetobacter sp. AS23 genome. Results of transcriptome analysis and biological tests suggested that steroid degradation pathway be the key pathway of AS23 to degrade TS. This study not only provides direct evidence that gut microbes mediate the rapid adaptation of herbivorous insects to phytochemical resistance, but also provides a theoretical basis for further research on the molecular mechanism of intestinal bacteria cooperating with pests to adapt to plant toxins.

## INTRODUCTION

Herbivorous insect species have specific preferences for host plants that produce resistant chemicals (1). These insects and plants have developed a relationship through co-evolution (2). Plants have evolved a range of insect-resistant mechanisms, and the selective pressure mediated by these mechanisms’ aids insect diversification (3–8). In addition to insect enzymes that can detoxify plant secondary metabolites (PSM), gut bacteria can also play a chemical-detoxifying role in insect–plant interactions (3, 9, 10). These microorganisms can promote digestion and contribute to PSM degradation (11, 12). The type and concentration of PSM can be important determinants influencing the composition of the gut microbiome (2, 13). The functions of the gut microbiome in the adaptations between phytophagous insects and plants have thus become the focus of research on insect–host plant interactions (3, 7, 8, 14, 15). The microbiome of a polyphagous insect gut can be affected by the presence of toxic PSM and be associated with the type of plant resistant compounds. (16, 17). Most host-specific phytophagous insects have a core group of microorganisms affected by the main PSM (2, 13, 18, 19). Host-specific insects are therefore good model organisms for studying the resistance of functional gut microbiome to specific chemicals.

Tea saponin (TS) of *Camellia oleifera* is triterpenoids that is toxic to seed-feeding weevil pest, *Curculio chinensis* (CW) (2, 20, 21). The TS content in different insect-resistant *C. oleifera* clones was negatively correlated with the damage level of CW (2, 20, 21). Studies have shown that over 220 insect species can feed on *C. oleifera*, and only CW is specialized seed feeder (19, 20). As host-specific to *C. oleifera*, CW’s infestations can decrease *Camellia* seed yield up to 60% (2, 20), and yearly economic losses in China exceed US $1.4 billion (22). The outbreak of CW is particularly serious and has hindered the sustainable development of local tea oil industry (22). Previous studies disclosed that various bacteria in the CW’s gut were associated with helping CW evade TS toxicity of *C. oleifera* (20). It was found that Acinetobacter sp. AS23 strain was the dominant strain in the gut microbiome of the surviving larvae with high resistance to host plants, and the abundance of AS23 strain was positively correlated with the TS content of host plants (21). Traceability analysis showed that the probability of the bacteria coming from soil was as high as 96% (21). However, plants have multiple anti-insect compounds, whether the specific enrichment of Acinetobacter is also related to other compounds. Furthermore, there was limit evidence of Acinetobacter sp. AS23 strain responsible for TS degradation, and the molecular mechanism of the degradation pathway has not been clarified.

To resolve these points, we intended to explore the relationship between plant toxin content and gut bacteria. By comparing the relative abundance, diversity, and composition of gut microorganism within weevils collected from different *C. oleifera* clones, we determined the specific PSM that was the main element affecting the gut microbiome. To study how gut bacteria degrade TS and to elucidate the corresponding molecular mechanism would uncover the role of the intestinal microorganisms during the process of CW invasion. We, thus, performed isolation and identification of the functional strain and investigated its relationship with TS degradation mechanism. Biological tests were carried out to verify the ability of functional bacteria to detoxify TS with CW larvae. The study aims to explain the mechanism of gut bacteria mediating CW adaptation to chemical resistance of *C. oleifera* clones from the molecular level and to reveal the role of microbial-dominated biological factors during pest outbreak, providing a new perspective understanding the mechanism of plant pest outbreak, which is of great scientific and potentially economic significance.

## RESULTS

### Fruit TS content affected the CW gut microbiome structure and enriched the bacteria functional for TS degradation.

**FIG 1:**
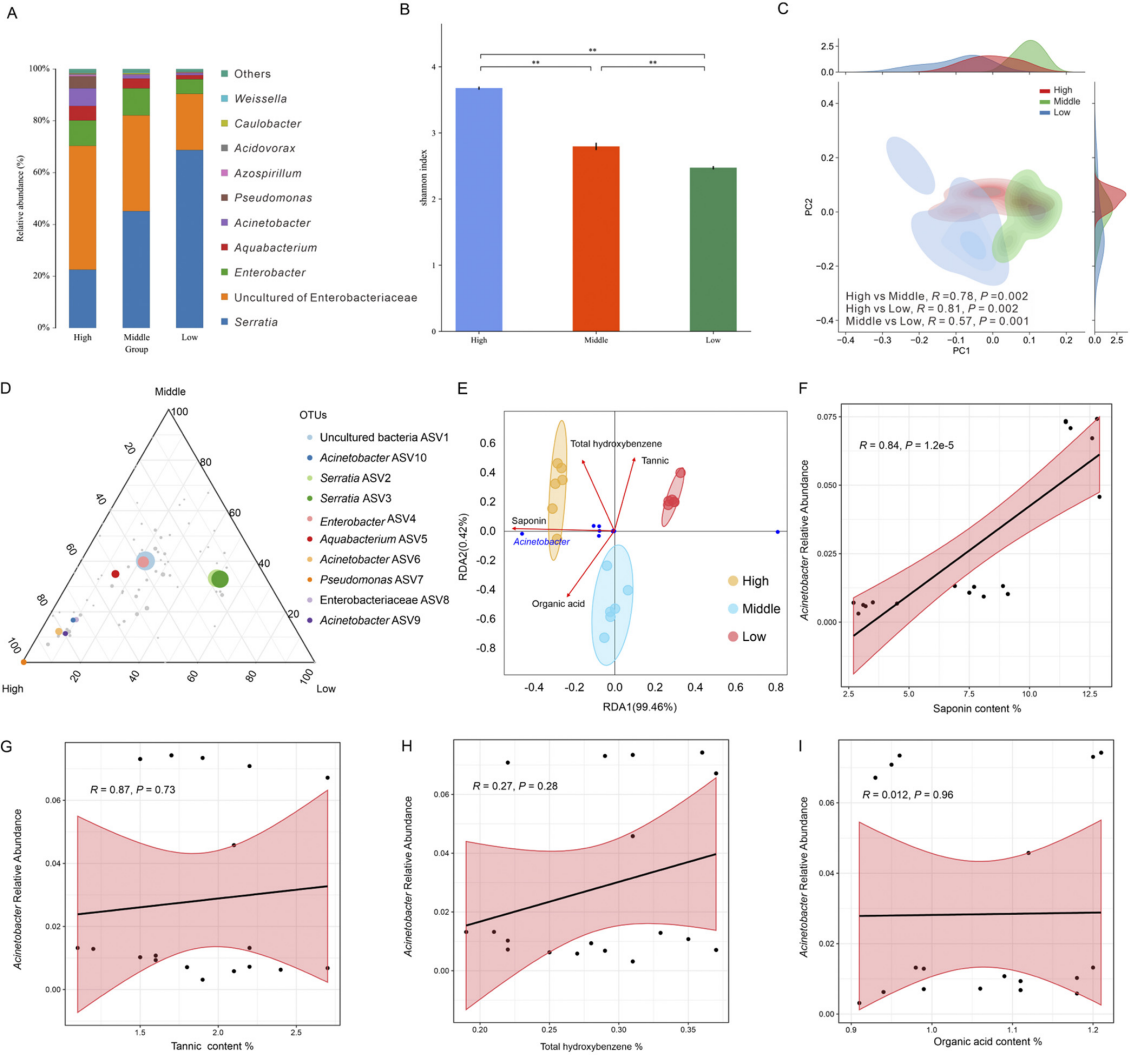
Gut microbial communities of *Curculio chinensis* larvae are affected by the types and concentrations of plant secondary metabolites. (A) Microbial composition of the three samples shown at the genus level. Shown are changes in the relative abundances of the 10 most abundant species (on average) in the High, Middle, and Low samples. (B) Shannon index analysis for three groups of samples, respectively. **, indicates a significant difference at *P = *0.01 between two groups. (C) Principal co-ordinate analysis was based on 16S rRNA gene weighted Bray–Curtis distances of these three groups respectively and used ANOSIM of variance, *P < *0.001. The 3 colors represent different sample sources (High, Middle, and Low). Different *R* values represent the level of difference (significant: *R *> 0.75, moderate: 0.75 >*R* >0.5). (D) Ternary plot showed all ASVs (>5%) found in CW larvae that fed on host plants with different resistance. Colored points represent ASVs that are enriched in one compartment. The grayish points represent ASVs that are not significantly enriched in a specific compartment. The size of the colored dots corresponds to the size of the abundance of these ASVs. (E) Redundancy analysis was used to resolve the relationship between the gut microbiota composition and the 3 levels of resistance in the host plants. These dots refer to the microbiota composition at the ASV level of each sample living on a host with high, middle, and low resistance, respectively. Blue dots represent the most abundant genus of gut microbiota, and red arrows symbolize four plant secondary metabolites. (F) (G) (H) and (I) Linear-regression analysis of (F) saponin, (G) tannin, (H) total hydroxybenzene and (I) organic acid with Acinetobacter relative abundance represents the relationship between these secondary metabolites and relative abundance of Acinetobacter.

To examine the relation between plant defense chemicals and key microbes metabolizing these toxic substances, we initially extracted genomic DNA from all samples and obtained 16S rRNA sequences. All high-quality sequences were then assigned to amplicon sequence variants (ASVs) with 100% similarity. Rarefaction curves of all samples for 16S rRNA gene sequencing tended to be saturated (Fig. S1), indicating that the sequencing depths for these specimens were appropriate. The relative abundance of the bacterial communities was shown, and the richness showed that the dominant gut bacteria at the genus level was diverse among CW feeding on the 3 selected clones (Fig. 1A). An uncultured genus of Enterobacteriaceae was predominant in the samples collected from the *Camellia* clone with high TS, while *Serratia* was predominant in clones with middle and low TS.

For community diversity analysis, the Shannon indices showed significant differences between the samples from host plants with high (3.6779 ± 0.023), middle (2.7964 ± 0.055), and low (2.474 ± 0.025) TS content (Fig. 1B). The Simpson index also showed significant differences between the groups (high versus middle, middle versus low, and high versus low). However, there were no significant differences in most alpha diversity indices (ACE, Chao1, and PD whole tree) as a group (high versus middle) (Table S1). Beta diversity (Fig. 1C) was tested using principal coordinates analysis (PCoA) based on Bray–Curtis’ dissimilarity distances. ANOSIM showed that weevils feeding plants with different resistance had significant differences in their bacterial community composition. The difference between the high and middle groups was significant (*R *> 0.75, *P = *0.002), and the difference between the high and low groups was also significant (*R *> 0.75, *P = *0.002). The difference between middle and low groups was moderate (0.75 > *R* > 0.5, *P = *0.001) (Fig. 1C). We showed the relative abundance of 10 high-abundant common ASVs (Uncultured bacteria ASV1, Acinetobacter ASV10, *Serratia* ASV2, *Serratia* ASV3, Enterobacter ASV4, *Aquabacterium* ASV5, Acinetobacter ASV6, Pseudomonas ASV7, Enterobacteriaceae ASV8, and Acinetobacter ASV9) in 3 groups (high, middle, and low) using a ternary plot (Fig. 1D). Acinetobacter ASV10, Acinetobacter ASV6, Acinetobacter ASV9, Pseudomonas ASV7, and Enterobacteriaceae ASV8 were significantly enriched in the guts of weevils exposed to the highly resistant host plants. Enterobacter ASV4 and uncultured bacteria ASV1 were relatively uniform in distribution among the 3 groups. *Serratia* ASV2 and *Serratia* ASV3 were relatively enriched in the low-treated insects. *Aquabacterium* ASV5 was relatively enriched in high-treated insects and middle-treated insects.

To determine the secondary metabolites associated with the involvement of the microbiome in detoxification, the relationship of plant secondary metabolite content and bacterial community composition were evaluated using the direct gradient analysis technique (RDA). High group was most likely related to TS content, and the abundance of Acinetobacter was closely related to the concentration of TS (Fig. 1E). This strongly suggested the role of TS affecting gut microbiome structure and the role of the intestinal bacterium Acinetobacter relating to TS metabolism. The linear-regression analysis of the content of 4 secondary metabolites (saponin, tannin, total hydroxybenzene, and organic acid), and the abundance of Acinetobacter showed that the relative abundance of Acinetobacter was closely related to the TS content, and there was a significant linear relation (*R*^2^ = 0.70941) (Fig. 1F, G, H, and I).

### TS-degrading bacteria belong to Acinetobacter and the functional strain AS23 genome contains many genes related to PSMs degradation.

**FIG 2:**
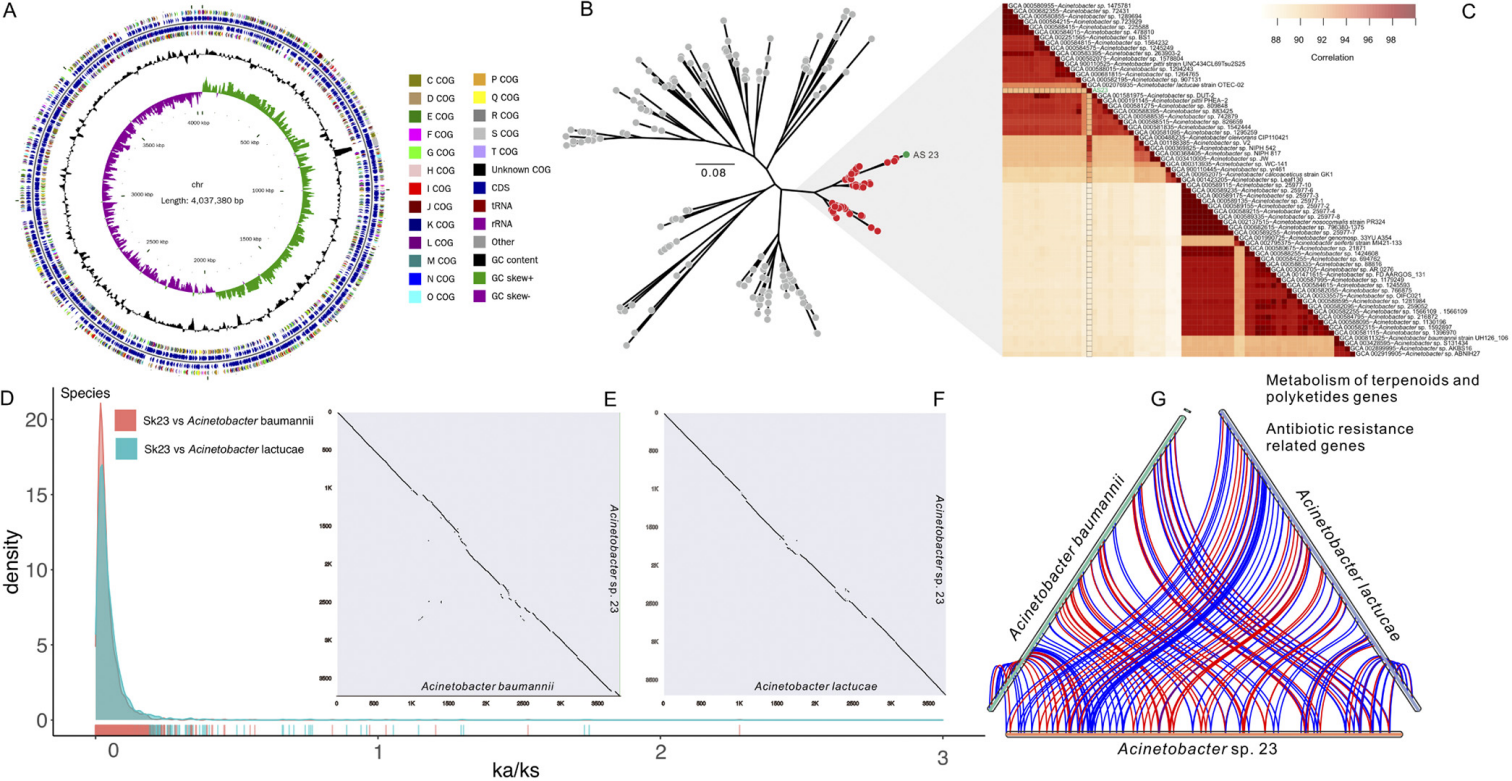
Species identification and analysis of AS23 strain based on genomic data. (A) Circular representation of the AS23 genome. From inner to outer: The first circle represents the scale; The second circle represents GC skew; The third circle represents GC content; The fourth and seventh circles represent the COG to which each CDS belongs; The fifth and sixth circles represent the positions of CDS, tRNA and rRNA contained in genome. (B) Genomic phylogenetic tree of the Acinetobacter sp. AS23 (marked in green), isolated from CW gut. (C) The heatmap on the right represents ANI values. The scale bar represents 0.08 substitutions per nucleotide position. And comparative genomic analysis of AS23 strain, A. baumannii and *A. lactucae*. (D) *K*_a_/*K_s_* density distributions for orthologous gene pairs between AS23 and other species (A. baumannii and *A. lactucae*). (E) and (F) The dot plots of orthologous genes in different species ([E] AS23 versus A. baumannii and [F] AS23 versus *A. lactucae*) were used to see pairwise synteny. (G) Syntenic analysis among AS23, A. baumannii and *A. lactucae*. A synteny block was constituted by one coding sequence. Genes associated with Antibiotic resistance and Metabolism of terpenoids and polyketides in chromosomes are highlighted in the figure.

Based on 16S rRNA sequence, we selected a pure single colony of Acinetobacter (AS23) from the gut of CW. After filtering the raw data of Illumina, we obtained 8,321,490 reads and 1,237,365,283 bp of high-quality data. In addition, we counted total sequence number (488,891) and length (4,611,716,664) of PacBio. We obtained a genome (4,037,380 bp) that consisted of a circular chromosome with no plasmid (Fig. 2A). The GC content of the genome was 39%. Based on coding sequence genes prediction, 3,736 ORF were obtained, and the total ORF length by gene prediction was 352,1808 bp. The genome encoded 74 tRNA genes and 35 ncRNA (Fig. 2A). A total of 18 rRNA genes were also present on the chromosome, including six 5S rRNAs, six 16S rRNAs, and six 23S rRNAs (Fig. 2A). To determine species groupings of the strain AS23 within Acinetobacter, we used the genomic sequences of AS23 and other 227 Acinetobacter species for phylogenetic analysis. Phylogenetic trees showed that AS23 was more closely related to *A. lactucae* than other species of Acinetobacter (Fig. 2B). But the ANI value between AS23 and *A. lactucae* was 91.277878%, less than 95% ANI cutoff values for bacterial species delineation (Fig. 2B). Therefore, we treat AS23 as Acinetobacter sp. AS23 (Fig. 2B).

The genetic distance and relationship between AS23 and other species were confirmed using the results of phylogenetic tree and the ANI values. We also calculated *K*_a_/*K_s_* values (Fig. 2D) of orthologous genes between the AS23 genome and the other 2 species (A. baumannii and *A. lactucae*), suggesting a negative selection (*K*_a_/*K_s_* < 1) of most gene pairs and a positive selection (*K*_a_/*K_s_* > 1) of a few gene pairs (AS23 versus *A. baumanni*: 4 pairs; AS23 versus *A. lactucae*: 6 pairs).To identify evolutionary relationships between the AS23 strain and other Acinetobacter species, we selected *A. lactucae* (genetically close) and the multidrug-resistant A. baumannii (genetically distinct) for genomic comparative analysis (Fig. 2E and F). The result showed a good synteny between AS23 and the 2 species. Based on CARD analysis and annotation of AS23 genome, the karyotype figure with the resistance genes and metabolism of terpenoids and polyketides genes mark showed that the resistance genes and metabolism genes of AS23 had good collinearity with A. baumannii and *A. lactucae* (Fig. 2G). We obtained 59 antibiotic resistance genes (41 antibiotic resistance genes, 21 antibiotic target genes, and 3 antibiotic biosynthesis genes) using the comprehensive antibiotic resistance database analysis. In addition, we found 47 metabolisms of terpenoids and polyketides genes using Kyoto Encyclopedia of Genes and Genomes (Table S3).

### The functional stain AS23 degrades TS and improves the CW larvae survival rate.

**FIG 3:**
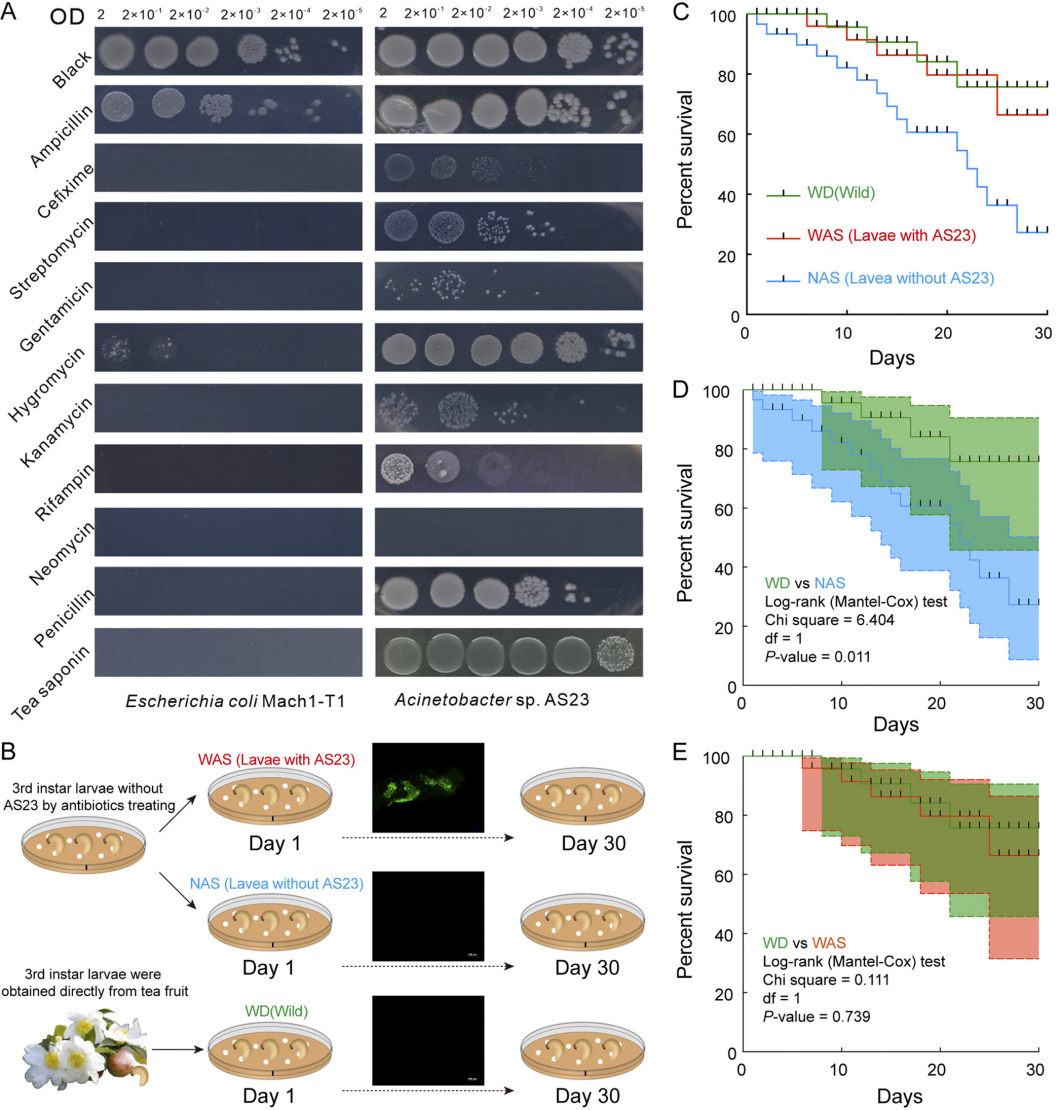
Antibiotics and saponin tolerance test of Acinetobacter sp. to TS. (A) Tolerance test of AS23 strain of tea saponin (5g/L) and 8 antibiotics. *Esherichia coli* was the control group and AS23 was the treatment group. (B) Experimental design to verify that AS23 helps CW larvae tolerate TS toxicity. (C) (D) and (E) Survival curves of tea saponin tolerance of CW larvae with different treatments. (C) Survival curves of CW larvae fed with 5g/L TS among 3 groups. (D) WD group vs WAS group. (E) WD group vs NAS group.

To further identify the tolerance of the AS23 strain to various antibiotics for the subsequent functional verification of the strain, we compared its tolerance to TS (5 g/L) and selected antibiotics (50 μg/L). The test showed that AS23 strain had varying degrees of resistance to 9 antibiotics and strong tolerance to TS (Fig. 3A). The strain AS23 grew on 6 gradients of media inoculated with ampicillin, hygromycin, and the TS on 5 gradients of media inoculated with penicillin and on 4 gradients of media inoculated with cefixime, streptomycin, kanamycin, and rifampin (Fig. 3A). Compared with E. coli, its resistance to gentamicin increased slightly (Fig. 3A). Noticeably, AS23 had no single colony growing at any level of the gradients inoculated with neomycin (Fig. 3A).

To further explore whether the screened strain can help CW larvae detoxicate TS, we compared the survival ratio of WD group (wild type), WAS group with AS23 in the gut and NAS group without AS23 on the feed containing tea saponin. The results showed that the survival curve of the WAS group with AS23 was highly overlapped with that of the WD group, and the difference was not significant by log-rank (Mantel-Cox) test (*P = *0.739) (Fig. 3E). The mortality rate of NAS without AS23 was higher, and the survival curve was significantly different from that of WD group (*P = *0.011) (Fig. 3D).

### AS23 functional for tea saponin degradation show different gene expression patterns and pathways.

**FIG 4:**
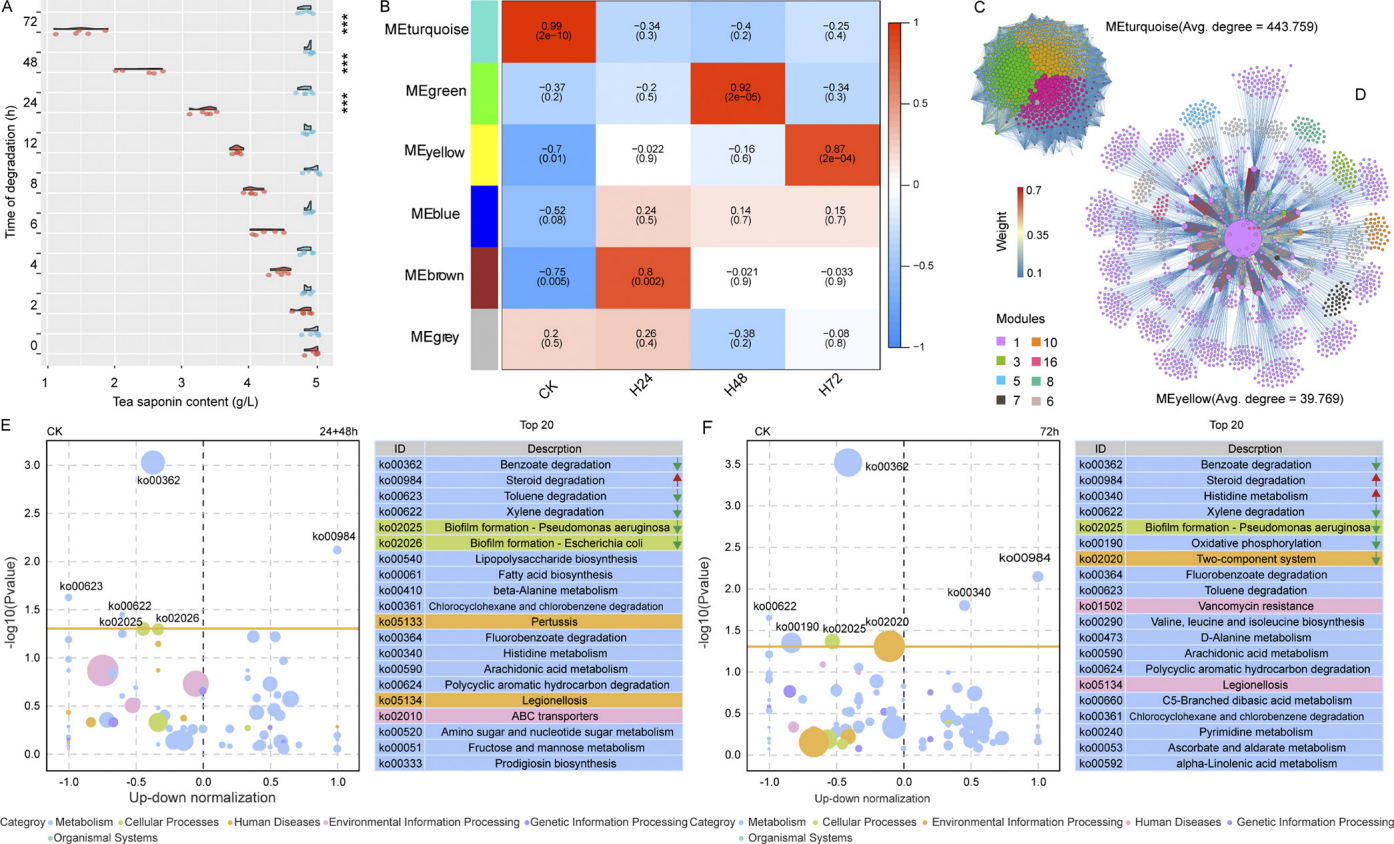
The differences of saponin content at different degradation times and weighted gene co-expression network analysis of different times. (A) Comparison between our functional bacteria AS23 (marked in red) and Sterile water (marked in blue). (B) Heatmap of the correlation between module eigengenes and degradation time. Each row corresponds to a module, and each column to a degradation time. Numbers in the figure report the correlations of the corresponding module eigengenes and degradation time, with the *P* values printed in parentheses. The degree of correlation is indicated by the color legend on the right side (red corresponds to positively correlated; blue corresponds to negatively correlated). (C) Genes network in the MEturquoise module (most relevant to CK). Node colors (magenta, yellow, and green) represent the different modules. The size of nodes is proportional to the degree. (D) Genes network in the MEyellow module (most relevant to H72). Node colors represent the different modules, and the size of nodes is proportional to the degree. The thickness of each edge between 2 nodes is proportional to the absolute values of Spearman's correlation coefficients. The colors of edges indicate the weight value. (E) Variation with the distribution of the 20 most abundant KEGG pathways between group 24 h + 48 h and CK. (F) Variation with the distribution of the 20 most abundant KEGG pathways between group 72 h and CK.

To confirm the degradation function of AS23 strain to TS, high-performance liquid chromatography (HPLC) was used to measure the content of TS at different times (2 h to 72 h) after adding TS medium. Compared with CK (bacteria solution with sterile water), significant differences began to appear at 6 h, and reached extremely significant level at 24 h (*P < *0.01) (Fig. 4A). The results showed that AS23 had significant ability to degrade TS, significantly at 24, 48, and 72 h (Fig. 4A). We then selected these 3 degradation times to further reveal the molecular mechanism of the degradation of TS based on transcriptome level (Fig. 4A). Weighted Correlation Network analysis (WGCNA) showed that each group (CK, H24, H48, and H72) was different in the most relevant module (Fig. 4B and C). The MEturquoise module was the most positively correlated with CK, the MEbrown module with H24, the MEgreen module with H48, and the MEyellow module with H72 (Fig. 4B). The modules significantly correlated with CK and H72 were selected for the visualization of gene network, and the results showed that the number of nodes and the weight values were different between these networks (Fig. 4C, D). The gene network of MEturquoise, the module most related to CK, was divided into 3 modules (green, yellow, and magenta), with an average degree value of 443.759 (Fig. 4C). In addition, the gene network of MEyellow, the module most related to H72, was divided into 8 modules, with an average degree value of 39.769 (Fig. 4D).

To further elucidate the functions of differentially expressed genes (DEGs) and to analyze the degradation mechanism of TS of AS23, KEGG pathway enrichment analyses between 24 h +48 h, 72 h and CK group were conducted by mapping to the KEGG database. The top 20 metabolic pathways associated with these DEGs in 24 h +48 h group were shown in Fig. 4E. Among these, the “Ko00984” pathway (steroid degradation) was significantly enriched in group 24 h +48 h (Fig. 4E). The top 20 metabolic pathways associated with these DEGs in 72 h group were shown in Fig. 4F. Among these, the “Ko00984” pathway (steroid degradation), Ko00340 (Histidine metabolism) were significantly enriched in group 24 h +48 h (Fig. 4F).

### Steroid degradation pathway could be the key pathway of AS23 to degrade TS.

**FIG 5:**
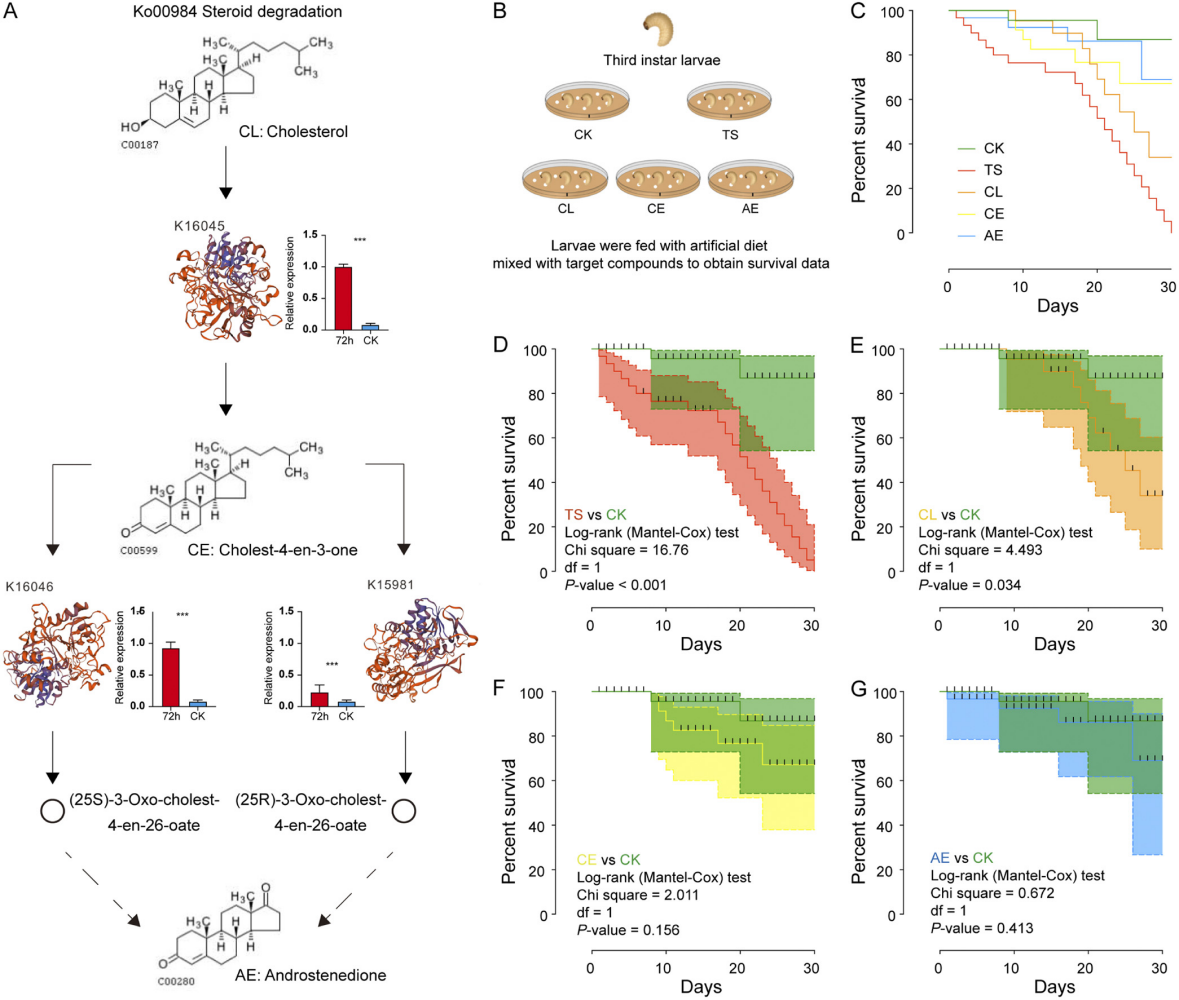
Function of steroid degradation pathway in TS degradation. (A) Ko00984 pathway associated with steroid degradation and RT-qPCR of genes annotated into K16045, K1046, and K15981. CK represents the bacteria solution of AS23 without adding TS liquid medium, and 72 h represents the solution after adding TS liquid medium for 72 h. (B) Experimental design to verify toxicity of 4 compounds. CK (toxin-free diet for larvae), TS (5g/L TS diet for larvae), CL (1g/L Cholesterol diet for larvae), CE (2.1g/L cholest-4-EN-3-One diet for larvae) and AE (6.4g/L Androstenedione diet for larvae). (C) (D) (E) (F) and (G) Survival curves of CW larvae with 4 compounds. (C) Survival curves of CW larvae among 5 groups. (D) TS group vs CK group. (E) CL group vs CK group. (F) CE group vs CK group. (G) AE group vs CK group.

To confirm the results obtained using KEGG, we chose 3 genes of Ko00984 (K16045, K1046, and K15981) and performed quantitative real-time PCR (qPCR). The results of the qPCR (Fig. 5A) showed that, compared with CK, the expression levels of the 3 genes were significantly increased in H72. According to the analysis of Ko00984 pathway, cholesterol (CL) was the catalytic substrate of K16045, and the product was cholest-4-en-3-one (CE) (Fig. 5A). CE was decomposed into androstenedione (AE) by K16046 and K15981 as the substrate (Fig. 5A). Based on this, the toxicity study results of TS, CL, CE, and AE compounds on CW larvae showed that the mortality rate of CK group without toxin was the lowest (Fig. 5C). The mortality of TS group was the highest (Fig. 5C and D), followed by CL group (Fig. 5C and E), indicating that the product CL still had certain toxicity to CW larvae. The survival curve showed little difference between CE and AE groups (Fig. 5F and G).

## DISCUSSION

Plants employ their secondary metabolites to defend against infestations of phytophagous arthropods (6). Our results showed that the *C. oleifera* clones with different levels of TS content can impact the structure and composition of the weevil gut microbiome (Fig. 1A and B). The difference was more significant when compared with TS-low group (Table S1). The calculated β-diversity and analysis of similarity tests demonstrated that the composition of the CW gut microbiome was significantly associated with the TS level of *C. oleifera* clones (Fig. 1C). Our result agrees with the study that caffeine concentration may alter *Hypothenemus hampei* gut microbiome composition (13). Other findings also disclosed that certain types of plant secondary metabolites can have an impact on the gut bacterial community (2, 6).

Host plants’ chemical defenses can have direct toxicity on herbivores insects. Chemical interaction with insect gut microbes, however, may enhance insect ability to contend with host plants defenses (7). RDA analysis between 4 secondary metabolites and 3 levels of TS content showed that saponin level was most correlated with the high group (Fig. 1E). The result is consistent with the previous study that the gut bacterial community treated by TS showed a significant gradient change associated with concentration in contrast to four other metabolites (2). We further confirmed that TS plays an important role determining the CW gut microbiome composition. Three Acinetobacter ASVs, 1 Pseudomonas ASV, and 1 Enterobacteriaceae ASV were enriched in the beetles that feed on TS-high clone (Fig. 1D), and TS content had significant linear relation with Acinetobacter abundance among the 4 metabolites (Fig. 1D, E, and F). Another study reported that Pseudomonas genus in the environment can increase the content of saponin in host plants (23). It is, thus, meaningful that Pseudomonas ASV7 was enriched (100%) at high and middle groups, but not present (0%) at low group. In addition, a previous study showed that Pseudomonas can promote hydrocarbon degradation in a saponin-rich environment (24). The plant endophyte Enterobacter within *Panax notoginseng* was related to saponin content and might be involved in the conversion of saponin (25). Although there is no direct evidence that Pseudomonas and Enterobacteriaceae participate in saponin degradation, a common Pseudomonas strain that can colonize plant roots has significant interactions with plants and induces plant growth (26, 27). Therefore, we speculate that Pseudomonas ASVs may influence the production of saponin in *Camellia* and Enterobacteriaceae ASVs may be related to the degradation of other PSM. Nevertheless, Acinetobacter has been considered as a key player directly involved in the degradation of saponin and other plant secondary metabolites (2, 6). Our finding here clearly demonstrated that Acinetobacter of the CW gut microbiome plays an important role in the TS-detoxification process.

A pure single strain was collected from CW gut and identified as Acinetobacter sp. (Fig. 2). The Acinetobacter species was divided into 4 Clades (Clade I, II, III, and IV) (26). The genomes of Acinetobacter pittii, *A. lactucae*, *A. oleivorans*, A. calcoaceticus, *A. nosocomiali*, *A. seifertii* and A. baumannii were included for phylogenetic analysis indicating AS23 resided in clade II (Fig. 2B) (28). To confirm the genetic difference between AS23 and similar species, we described the genomic collinearity and *K*_a_/*K_s_* values (Fig. 2D). In this study, the ANI values between AS23 and some species from “Acb clade” were higher, indicating AS23 belonging to “Acb clade” (Fig. 2C). Interestingly, the Acb complex members often have the higher number of unknown genes commonly associated with recent HGT (horizontal gene transfer) (29, 30). This showed that most of the paralogous genes were under purifying selection, confirming the slight differences among genes responsible for antibiotic resistance and metabolism of terpenoids and polyketides. Phylogenetic analysis results disclosed that the genetic distance between AS23 and *A. lactucae* was closer, which is the same to that of genomic collinearity analysis (Fig. 2E and F). Notably, *A. lactucae* and A. baumannii were reported as members of the “Acb clade” (29) consisting of at least 7 species (A. baumannii, A. calcoaceticus, *A. lactucae*, A. nosocomialis, *A. oleivorans*, A. pittii, and *A. seifertii*) (28). The genome published here, along with the assessment of antibiotic resistance and TS tolerance, provides a starting point for our studies on gut bacterial degradation of TS ability.

Tolerance of Acinetobacter sp. AS23 to TS and antibiotics was, thus, evaluated, and the number of colonies growing in 9 different antibiotic and TS media were determined (Fig. 3A). For the treatment group, this strain showed strong tolerance to TS and high antibiotic tolerance except to gentamicin and neomycin. Noticeably, AS23 was completely intolerant to neomycin, which might be due to the absence of the plasmid compared with A. baumannii (31). Another study disclosed that the antimicrobial resistance of bacteria was related to their different living environments (humans or soil), e.g., A. baumannii (28). It has also been reported that bacteria have experienced genome reduction through gene loss events when the certain strain undergoes adaptive specialization for a new niche during evolution (32). Antibiotics can be used to destroy pests by affecting the abundance of the Acinetobacter in the gut (33), and it provides a possible new strategy for pest control. Furthermore, the determination of the tolerance of different antibiotics provides a theoretical basis for the selection and application of antibiotics in the subsequent test of this strain. On this knowledge, we obtained AS23-free CW larvae (Fig. 3B). The larva was used to connect back to AS23 strain to verify whether the strain helped the larva to tolerate tea saponin (Fig. 3B). The results showed that the survival rate of the larva containing AS23 in the intestinal tract was basically the same as that of the wild type (Fig. 3C, D, and E), suggesting that AS23 strain play a role relating to tea saponin toxicity in the larval intestine (Fig. 3C, D, and E).

The follow-up is, thus, to demonstrate whether AS23 can degrade TS. According to the TS residual content determined by HPLC, the content of TS decreased sharply after 24 h of degradation (Fig. 4A). Based on transcriptome sequencing and WGCNA analyses, it was found that the modules with the highest correlation were different at different degradation times (Fig. 4B, C, and D). We speculated that there might be different types of functional genes playing a key role at different degradation time points. The key pathway Ko00984 and 3 key genes K16045, K16046, and K15981 related to TS degradation were further identified (Fig. 4E and F). Saponins are divided into terpenoid and steroid saponins, among which TS belongs to pentacyclic triterpenoid saponin, and there is no steroid in its chemical structure. We found that Ko00984, a pathway relating to steroid degradation, was significantly enriched during TS degradation by AS23. This indicated that Ko00984 is related to TS degradation, but the specific process from TS to steroids is not known yet.

We further provided insight into the TS degradation function and degrading genes of AS23 using the RNA-seq. One KEGG pathway and 3 degradation-related genes were identified in H72 (Fig. 5A). This provided a resource for adaptation mechanism of the weevil to PSM although further research is needed to explore the function of the identified degrading genes (Fig. 5A). The transcriptome has been used to identify specific genes that associated with degradation function in bacteria (34, 35). In the study (34), the degradation mechanisms of long-chain n-alkanes, the transcriptomes of the Acinetobacter pittii strain SW-1 grown in C_20_ or CH_3_COONa (NaAc) as the sole carbon source were compared. A total of 437 DEGs were upregulated, and the transcriptional levels of *alkB* increased 78.28-fold (34). The other study dealing with phosphorus (P) adsorption mechanisms in a phosphate-solubilizing bacterium (PSB) Acinetobacter sp. M01, genes for phenylalanine, amino acid, and glycerol lipid metabolisms were identified in M01 DEGs (35). In addition, our study on the toxicity of the compounds involved in Ko00984 pathway to CW larvae further confirmed that steroid degradation pathway may be the key pathway of AS23 to degrade TS (Fig. 5C, D, E, and F). Thus, we concluded that the key genes of AS23 involved in TS degradation may be enriched in Ko00984, which are involved in the regulation and expression of 3 important oxidases.

Overall, we demonstrated that a host-specific pest CW possesses a gut microbiome that is significantly associated with TS degradation (36). We also demonstrated that the Acinetobacter genus from the CW gut microbiome has significant linear relation with the TS content in *Camellia* fruits. Acinetobacter species plays an important role in the digestive tract of many insects, contributing to nutrition utilization, life cycle completion and protecting from the host plants secondary metabolites and unstable environmental conditions (17, 33, 37–39), and have been reported widely from insect, animal, and human hosts (39, 40). The role of Acinetobacter in the gut microbiome of CW demonstrated here helps us understand the plant secondary metabolite metabolism and select biocontrol strategies where the dominant gut bacteria associated with the core PSM are targets.

## MATERIALS AND METHODS

### Sample collection and processing.

This study ensured that compliance with ethics standards were met. On October 5, 2019, CW larvae were collected at the Jianshan plantation in Qingtian, Zhejiang, China (28°11′51.61″ N, 120°23′15.25″ E). Larvae were transported to the laboratory within the fruits of 3 *C. oleifera* clones (Chang-lin 40, Chang-lin 3, and Chang-lin 53), which have different levels of TS content (high, middle, and low), respectively. The fruits were marked and placed in plastic crates, and the larvae were reared in sterile soil to obtain adults the next year for subsequent inoculation tests. Plants (*n* = 10) of each clone were randomly selected as samples (41). These plants were sealed with plastic mesh to prevent the oviposition of wild CW. After pupating in sterile soil, 10 pairs of adults (male:female = 1:1) reared indoors were introduced to original orchards on each plant and used to mate. Based on the heights and geographical locations of fruits from the same plant, fruits of the 3 clones were randomly selected at 3 different levels (upper, middle, and lower) and 4 directions (east, south, west, and north). For each clone, 20 fruits were collected in every level of different directions and 240 fruits were collected totally. Fully developed live adults were randomly selected and dissected in phosphate-buffered saline (0.1 M, PH = 7.2) when they emerged from seeds. After dissection, the gut of each 3 larvae were placed into a sterile centrifuge tube. For each group (high, middle, and low), the gut of 9 larvae as a replicate and 6 replicates were set, and a total of 54 larvae were used to construct the subsequent sequence library.

### Detection of four toxic secondary metabolites in *C. oleifera* seeds.

Seeds from each clone were ground into powder using a grinder (model: 800C; 30000 rpm/min). Seed powder was filtered using an 80-mesh sieve and the final weight was 0.5 g. TS content was determined as described by Zhang et al. (2). The chromatographic conditions were as follows: chromatographic column, Eclipse XDB-C_18_ (4.6 mm by 250 mm, 5 μm; Agilent Technologies, Inc.); mobile phase, methanol-water (9:1); detection wavelength, 210 nm, and a column temperature of 25°C. The standard (TS standard, weighed at 0.5 g), was diluted with methanol using ultrasound, and adjusted to a volume of 100 mL in a volumetric flask. Then, 1.00-, 3.00-, 5.00-, 7.00- and 9.00-mL amounts were removed and dissolved in 5 volumetric flasks (50 mL) in a constant volume of methanol. The standard was filtered using a microporous membrane (0.45 μm), and the regression equation obtained by using the mass concentration and the corresponding peak area as the *x*, *y* coordinates, respectively. For the specimens, TS content was measured as follows: TS (0.058 g) was sampled and dissolved in methanol via ultrasound; it was then diluted in a 50 mL volumetric flask, filtered through a microporous membrane (0.45 μm), and then subjected to HPLC. TS content (%) was = X * V/m * 100, where X is the concentration of the sample solution (g/mL), V is the volume of the sample at constant volume (mL), and m is the mass of the sample (g).

Tannin acid content was determined following the methods of Pan et al. (42) and Folin et al. (43), with some modifications. The fresh sample (0.5 g) was ground, weighed, and put into a conical flask. Then 70% ethanol (20 mL) was added. This flask was placed in a constant temperature water bath to reflux at 90°C for 15 min. After cooling, the residue was extracted three times with 70% ethanol (20 mL). The filtrate was combined into a volumetric flask (100 mL) with 70% ethanol and shaken well. The fluid under test (5 mL) was put into a volumetric flask (50 mL) containing water (25 mL) and the phosphotungstic phosphomolybdic acid reagent (2.5 mL) and saturated Na_2_CO_3_ solution (10 mL). Each sample was placed in a 100-mL flask and was brought to volume with distilled water, and then the flask was shaken. Based on the absorbance determination, the tannic content was measured at 680 nm, and the standard curve was drawn.

Total hydroxybenzene content was detected by the method of Bekir et al. (44) with minor modifications. Gallic acid was selected as the standard for compound identification. A 0.5 mL diluted sample was placed and extracted in a centrifuge tube (10 mL), and 2.5 mL folin reagent (0.2 mol/L) was added. The mixture was allowed to stand for 5 min, and 2 mL Na_2_CO_3_ solution (75 g/L) was then added and mixed well. After incubating the mixture for 1.5 h in the dark, absorbance was measured at 765 nm using an UV spectrophotometer (UV-1900i, Japan). The blank control was set from which distilled water replaced the Na_2_CO_3_ solution. Based on the standard curve associated with total hydroxybenzene, the content was calculated, and the results were expressed as mg/g. A total of 18 samples were analyzed, and each sample was analyzed three times.

Organic acid content was measured following the method of Tomar (45). Amount of 3 mL of each supernatant was filtered into a 5-mL bottle for chromatographic analysis. Based on chromatographic analysis, the parameters were set as follows: column, Agilent TC-C18 (250 mm × 4.6 mm, 5 μm); detector, VWD; mobile phase, methyl alcohol, and 0.01 mol/L KH_2_PO_3_ (3:97) (pH adjusted to 2.80 with phosphoric acid); detection wavelength, 210 nm; flow rate, 1 mL/min; injection volume, 10 μL, and column temperature, 25°C. The standard solution (organic acid standard reserve solution, diluted 10-fold) was used to continuously inject six times, and we obtained the precision, which was calculated by the peak area. Each clone was precisely weighed in 6 fractions (10 g each), pretreated, and chromatographically analyzed to calculate the relative standard deviation. Two extracts of the same organic acid were prepared (5 mL each); one extract was used as the background and 5 mL of organic acid standard solution was added to the other. After pretreatment, chromatographic analysis was performed to determine the standard recovery rate of each organic acid. A total of 18 samples were analyzed, and each sample was analyzed three times.

### DNA extraction and high-throughput sequencing of gut microbiome.

The CW gut specimens were extracted by the method of Zhang et al. (2) with minor modifications. These specimens were soaked using 75% alcohol for 1 min, and then the gut tissues were placed in phosphate-buffered saline (1×PBS) and dissected. The collected tissues were homogenized with a fixed ratio of 1 tissue to 4 PBS. The CW from the 3 *C. oleifera* clones were selected, and their gut specimens were combined and homogenized. The homogenate was centrifuged at 7500 rpm, and the supernatant was placed into a new tube before total DNA extraction of the gut microbiome. We used the bacterial DNA extraction method of the Qiagen DNeasy blood and tissue kit. The DNA concentration was determined, and the DNA size was assessed. PCR amplification of the bacterial 16S rRNA gene V3–V4 region was performed using the forward primer 341F (5′-CCTAYGGGRBGCASCAG-3′) and the reverse primer 806R (5′-GGACTACNNGGGTATCTAAT-3′) (2). Pair-end 2 × 300 bp sequencing was performed using the Illlumina MiSeq platform with MiSeq reagent kit v3 (600 cycles) at Personal Biotechnology Co., Ltd (Shanghai, China).

### Diversity detections statistical analysis.

Raw paired-end reads of 16S rRNA genes obtained in this study were deposited in the National Center for Biotechnology Information (NCBI) database under the accession number PRJNA756472. Microbiome bioinformatics were performed with QIIME 2 v2019.4 (46) with slight modification according to the official tutorials (https://docs.qiime2.org/2019.4/tutorials/). Briefly, raw sequence data were demultiplexed using the demux plugin, followed by primers cutting with cutadapt plugin (47). Sequences were then quality filtered, denoised, merged and chimera removed using the DADA2 plugin (48). Non-singleton amplicon sequence variants (ASVs) were aligned with mafft (49) and used to construct a phylogeny with fasttree2 (50). The alpha diversity indexes (Shannon, Simpson, ACE, Chao1, and PD whole tree) calculated by QIIME2 were used to compare the gut bacterial diversity among clones. The beta diversity index was analyzed with a PCoA based on the Bray–Curtis’ distance matrix (51). Significance tests of the bacterial community from 3 clones were performed by analysis of similarities (ANOSIM) (52). The common ASVs among the 3 clones were shown in a ternary plot and the top 10 enriched ASVs were marked. Redundancy analysis (RDA) were performed using an online website (https://www.genescloud.cn/chart) to investigate the effects of toxic secondary metabolites on community composition (51). Linear-regression analysis and construction of the plot illustrating the relationship between the bacterial abundance and the content of 4 secondary metabolites were performed using R v4.0.4 (51).

### Isolation and identification of bacteria in the larval gut.

The larval gut specimens were placed in centrifuge tubes and mixed with liquid NA medium (5 mL), including peptone (5 g), beef extract (3 g), and NaCl (5 g) per L. To activate the bacterial fluid of the mixed bacteria, the tubes were placed in a shaking table for 24 h, and the parameters were 37°C and 200 rpm. After activation of culture, the activated bacterial fluid (10 μL) was evenly spread on disposable sterile culture dishes containing solid NA medium made by adding 15 g agar powder to each L of liquid medium. These culture dishes were placed in an incubator at a constant temperature (37°C) for 48 h. Single colonies were picked from these culture dishes and separated to pure single colonies on appropriate solid NA medium and placed in an incubator for 48 h. The edges of each pure single colony were dipped with a sterile needle, and then inoculated into liquid NA medium (5 mL) and placed in a centrifuge tube (15 mL) to activate them. After activation in a shaker for 24 h, 1 mL of bacterial fluid was sampled and used for DNA extraction using the TIANamp Bacteria DNA kit. The universal bacterial 16S rRNA primers (8F: 5-AGAGTTTGATCCTGGCTCAG-3′; 926R: 5-CCGTCAATTCCTTTAAGTTT-3′) were used to perform a 25-μL PCR (2). The PCR products were then checked with 1.0% agarose gel electrophoresis and sent for paired-end sequencing to obtain the 16S rRNA gene sequence for each single colony. BLASTN suite of Basic Local Alignment Search Tool (https://blast.ncbi.nlm.nih.gov/Blast.cgi) was used to assign the genus level of the isolates and the database title was Nucleotide collection (nt). Classified into different genera, the bacterial fluid of pure single colonies was placed in a sterile 40% glycerin solution and stored at −80°C.

The strain AS23 was obtained from a single colony isolated from the gut samples of CW and identified as an Acinetobacter sp. The DNA concentration was determined using the Quant-iT PicoGreen dsDNA assay kit and the size was assessed by bioanalyzer and gel electrophoresis. A total of 2 libraries were constructed, and the 2 libraries were sequenced using next-generation Sequencing (NGS) based on Illumina NovaSeq and Single Molecule Real Time Sequencing (SMRT Sequencing) based on PacBio Sequel, respectively. Based on Illumina NovaSeq, the extracted DNA from the strain AS23 was diluted for library preparation and the library was constructed with the TruSeqTM DNA Sample Prep kit following the Illumina TruSeq DNA Sample Preparation Guide. Based on 20 kb Template Preparation Using BluePippin Size Selection, a library for PacBio was constructed using the Template Prep kit 1.0, and the binding sample was fixed to zero-mode waveguides. Library construction and sequencing were performed by the Shanghai Personalbio Technology Co., Ltd.

The AS23 genome was sequenced using an Illumina platform (NovaSeq) to obtain paired ends of 150 base pairs (bp) with 400-bp inserts. The quality score profile of each sample was checked using FastQC v0.11.7 (53). The deplaning data obtained by an PacBio platform (Sequel) were assembled by HGAP v4 (54) and CANU v1.7.1 (55) to obtain the contig sequence. The high-quality reads of the Illumina NovaSeq were corrected using pilon software v1.18 (56) by the contig, and then the complete sequence was obtained after assembly. Circlator software v1.5.5 were used to confirm the circular chromosome. The coding sequences were predicted using GeneMarkS v4.32 (57). The tRNA genes in the whole genome were predicted using tRNascan-SE v1.3.1 (58). The rRNA genes were predicted using Barrnap v0.9 and the remaining non-coding RNAs were obtained by comparison with the Rfam database v14.1 (59). Phylogenetic analysis of genomes residing in the genus of Acinetobacter and AS23 was performed using PhyloPhlAn v3.0.60 (60). Genomic reference sequences of the 227 species of Acinetobacter were downloaded using the following options: “-g g_Acinetobacter -n 1”. The phylogeny was built using PhyloPhlAn with the following options: “–diversity high –fast”. In addition, ANI values were calculated using the FastANI v1.33 (https://github.com/ParBLiSS/FastANI) (61).

Genome sequences of Acinetobacter baumannii strain ab736 and *A. lactucae* ASM1312213v1 were downloaded from NCBI. MCscan (Python version) was used to search pairwise synteny between AS23 and these genome sequences, respectively (https://github.com/tanghaibao/jcvi/wiki/MCscan-Python-version), taking one gene as a putative syntenic region. Genes related to antibiotic resistance in the AS23 genome were obtained by CARD analysis (62). AS 23 metabolism genes of terpenoids and polyketides were found using Kyoto Encyclopedia of Genes and Genomes (63). The online version of KEGG (http://www.genome.jp/kegg/) was used to KEGG annotation of coding genes (63). Data set used “for prokaryotes” and method used bi-directional best hit (BBH).

Collinearity gene pairs which had these genes were marked, including Antibiotic resistance genes and Metabolism of terpenoids and polyketides genes. The respective orthologous genes between AS23, ab73, and ASM1312213v1 were used to assess the selection pressure of AS23 versus A. baumannii and AS23 versus *A. lactucae*. These orthologous genes were determined by collinearity gene pairs marked in synteny analysis, and these pairs was found by MCscan (Python version). Yn00 of the PAML package was used to calculate nonsynonymous and synonymous substitution (*K*_a_/*K_s_*) values (64). Ka and *K_s_* substitution rates were calculated the Yang and Nielsen method (YN method). MAFFT v7.480-1 was used to perform the multiple sequence alignment and ParaAT v2.0 was used to convert the file to paml format (65, 66). The yn00. ctl2 file was uploaded into the yn00 program and paml-yn00-run-pipeline was used for the analysis (https://github.com/LQHHHHH/paml-yn00-run-pipeline).

### Analysis of TS degradation ability of AS23 strain.

Before verifying the tolerance of AS23 strain to tea saponin, the tolerance of the strain to different antibiotics should be explored to prepare for subsequent aseptic operations. Escherichia coli Mach1-T1was introduced as a control to demonstrate the tolerance of AS23 strain. Single colonies of AS23 and E. coli were inoculated in a tube with 1.5 mL YEP liquid medium (Beef extract 10 g, yeast extract 10 g, and NaCl 5 g per L), respectively, and incubated overnight at 37°C at 200 RPM. After activation of culture, 200 μL AS23 and E. coli were added to a new tube with 10 mL YEP. The tube was then incubated until the OD_600_ value was equal to 2. We serially diluted the liquid to 2*10^−5^ according to the OD_600_ value. After that, the bacterial fluid (5 μL) of AS23 and E. coli were spread on sterile disposable plates containing liquid YEP medium with TS (5 g/L) and the antibiotics (50 μg/mL) of ampicillin, penicillin, streptomycin, kanamycin, gentamicin, neomycin, hygromycin, cefixime, and rifampin, respectively. The selection of these antibiotics was based on previous drug resistance studies of a well-known drug-resistant species, A. baumannii (29). There were 3 repetitions, and the final concentration of these antibiotics was 50 μg/mL. The plates were placed in an incubator at 37°C overnight.

On this basis, we fed CW larvae to the 3rd instar with AS23 intolerant antibiotic mixture feed (diet supplemented with a mixture of 50 μg/mL rifampin, 50 μg/mL neomycin, and 50 μg/mL gentamicin). Sixty larvae treated with antibiotics were divided into 2 groups: WAS group (reconnecting AS23 strain to gut) and NAS group (gut treated with sterile water). At the same time, 30 3rd instar larvae of CW in wild tea fruit were collected as WD group (wild weevils). Larvae in WAS, NAS, and WD groups were fed a diet containing 5g/L TS for 30 days. Three replicates were set for each group. Daily observation of deaths, data for survival analysis by GraphPad 8.0.

### Prokaryotic transcriptome analysis of TS degradation by AS23 strain.

Determination of TS content at different degradation times used liquid medium with TS as the single carbon source (5g TS with 99% purity, 5 g (NH4)_2_SO_4_, 2.5 g Na_2_CO_3_, 0.5 g MgSO_4_, 0.3 g KH_2_PO_4_, and 0.05 g FeSO_4_·7H_2_O) which was selected as the initial concentration for verifying the degradation ability of AS23 strain to TS (2). Bacterial solution of AS23 (10 μL, OD = 2) was absorbed into TS liquid medium (100 mL) and used for sampling in the treatment group. The bacterial solution was replaced with sterile water in the control group. Samples (*n* = 3) were taken at 0, 2, 4, 6, 8, 12, 24, 48, and 72 h, respectively. And then the TS content was determined by HPLC.

The prokaryotic transcriptome of AS23 degradation of TS at different times (24, 48, and 72 h) and solution without TS medium (CK) were determined, and the key genes at different times were compared and analyzed. Total RNA was extracted using TRIzol Reagent (Ambion), and was checked using 1% agarose gels before the quality of the RNA was assessed using an Agilent 2100 Bioanalyzer system. The RNA with a RIN value greater than 7.0 was used to produce the transcriptome library. The Zymo-Seq RiboFree Total RNA Library Kit was used to remove the rRNA from the total RNA. RNA sequencing libraries were generated using an NEBNext Ultra Directional RNA library prep kit for Illumina. Library quality was assessed on an Agilent Bioanalyzer 2100 system. The sequencing library was then sequenced with NovaSeq 6000 platform (Illumina) by the Personal Biotechnology Co., Ltd, and 150 bp paired-end reads were generated. The quality information of raw data in FASTQ format was calculated and then the raw data was filtered using Cutadapt v1.15. Clean data were obtained by removing reads containing adapters, reads containing poly-N, and low-quality reads. All subsequent analysis were based on high-quality clean data. Bowtie2 v2.2.6 (http://bowtie-bio.sourceforge.net/index.shtml) was used to map the reads to the reference genome. The level of gene expression was estimated by the expected number of fragments per kilobase of the transcript sequence per million base pairs sequenced (FPKM). We first performed cluster analysis on all samples according to the results of all gene expression levels. We compared CK with H24, H48, H72 and determined DEGs using R package DESeq2 (Parameter: Log_10_(Foldchange)=2, *P < *0.05) (http://www.bioconductor.org/packages/release/bioc/html/DESeq2.html). Ternary plots were used to analyze the distribution of the top 10 genes with relative expression levels in CK, 24 h + 48 h and 72 h groups. The number of genes specifically enriched in each group was calculated in Upset plots. Subsequently, the Kyoto Encyclopedia of Genes and Genomes (KEGG) pathway annotations were performed for these DEGs. Based on the KEGG functional annotation, we selected the pathway with the highest “RichFactor,” and then selected the key functional genes annotated in this pathway.

WGCNA was used to construct the gene co-expression network to efficiently mine the sequencing data, classify the data, and identify the hub genes. We first performed cluster analysis on samples based on Euclidean distance according to the results of all gene expression levels. Based on the gene expression level, the TOMSimilarity module was used to calculate the co-expression similarity coefficient between genes. The pickSoftThreshold function of the WGCNA package was used to weighted calculation (Parameter: power = 5). According to the parameter selected above, a weighted co-expression network model was built to classify genes. The correlation coefficients and *P*-values were calculated based on the expression profile characteristics of the degradation times and the module characteristic genes, and then the modules related to the different degradation times were screened. Finally, the modules most related to each degradation times (*P*-values < 0.05, and correlation coefficients ≥ 60%) were selected, and the genes network of the corresponding module was visualized using Gephi v0.9.2.

### Key pathway of TS degradation and toxicity analysis of degraded products.

We used qPCR to compare the expression levels between CK and 72 h. Primer BLAST (https://www.ncbi.nlm.nih.gov/tools/primer-blast/) was used for primer design, and the primers screened were as follows: K16045: F:5′-CGGCACAATGGCTGAAAACA-3′, R: 5′-TCCAGAACCAAACCAACGCT-3′; K16046: F:5′-TATCGGGCTAGGCCAGAGTT-3′, R: 5′-GGCGTTAAAACCTGTTGCCA-3′; K15981: F:5′-TGGCTATCGCGTTACAGTTCA-3′, R: 5′-TCCTGCAAACACGACACCTT-3′. A total number of 150 third-instar larvae were divided into 5 groups: CK (toxin-free diet for larvae), TS (5g/L TS diet for larvae), CL (1g/L Cholesterol diet for larvae, the concentration was set according to the concentration of Cholesterol in liquid phase after tea saponin degradation for 72 h), CE (2.1g/L cholest-4-EN-3-One diet for larvae), and AE (6.4g/L Androstenedione diet for larvae).

### Data availability.

Accession codes: 16S rRNA sequences have been deposited in the Sequence Read Archive (SRA) database with the accession code PRJNA756472 (SRR15534467 to SRR15534466). The AS23 genome raw data obtained in this study were deposited in the National Center for Biotechnology Information (NCBI) database with the accession number PRJNA756719.
