# Supplementary material for: Specific Enriched Acinetobacter in Camellia Weevil Gut Facilitate the Degradation of Tea Saponin: Inferred from Bacterial Genomic and Transcriptomic Analyses
Source: Microbiol Spectr. 2022 Nov 22;10(6):e02272-22. doi: 10.1128/spectrum.02272-22 (PMC9769793; doi:10.1128/spectrum.02272-22)
Supplement: Supplemental file 1 — Supplemental material. Download spectrum.02272-22-s0001.pdf, PDF file, 1.1 MB [file spectrum.02272-22-s0001.pdf]

Supplementary Information

Specific enriched *Acinetobacter* in *Camellia* weevil gut facilitate the degradation of tea saponin: inferred from bacterial genomic and transcriptomic analyses

Zikun Li[a,b], Suya Huang[a,d], Xinghua He[c], Haijie Ma[d], Xudong Zhou[a,b], Haiping Lin [a,b,*], Shouke Zhang[a,b,*]

[a] State Key Laboratory of Subtropical Silviculture, Zhejiang A & F University, Hangzhou 311300, People's Republic of China

[b] College of Forestry and Biotechnology, Zhejiang A & F University, Hangzhou 311300, People's Republic of China

[c] Zhoushan Academy of Forestry Science, Zhoushan 316000, People's Republic of China

[d] College of Horticulture Science, Zhejiang A & F University, Hangzhou 311300, People's Republic of China

***Correspondence to**: Prof. Haiping Lin and Dr. Shouke Zhang, College of Forestry and Biotechnology, Zhejiang A & F University, Hangzhou, China. Email: socrazhang@zafu.edu.cn

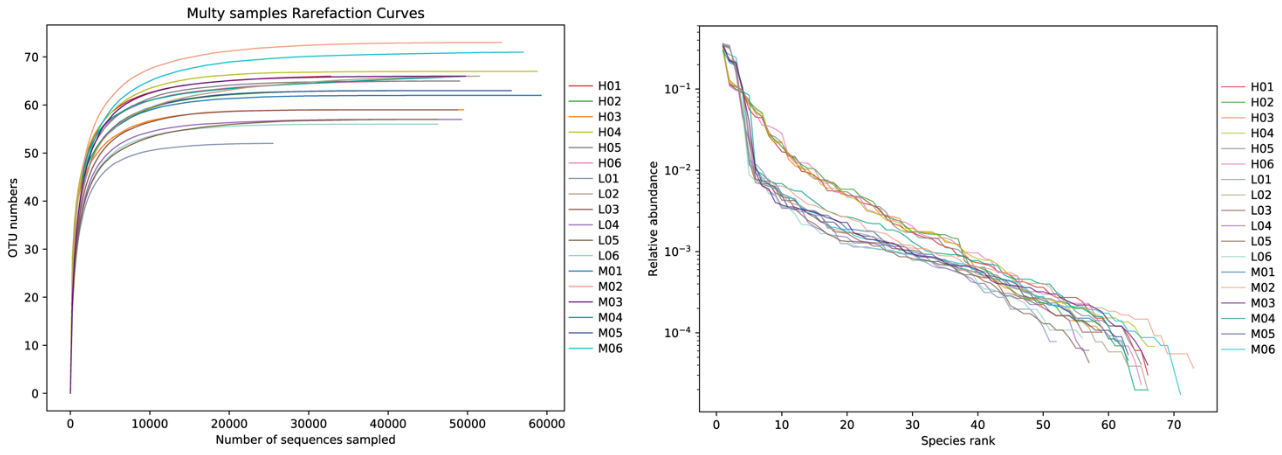

Fig. S1. Bacterial rarefaction curves are used to assess the depth of coverage for multi samples. (Multi samples were distinguished by different colors of lines)

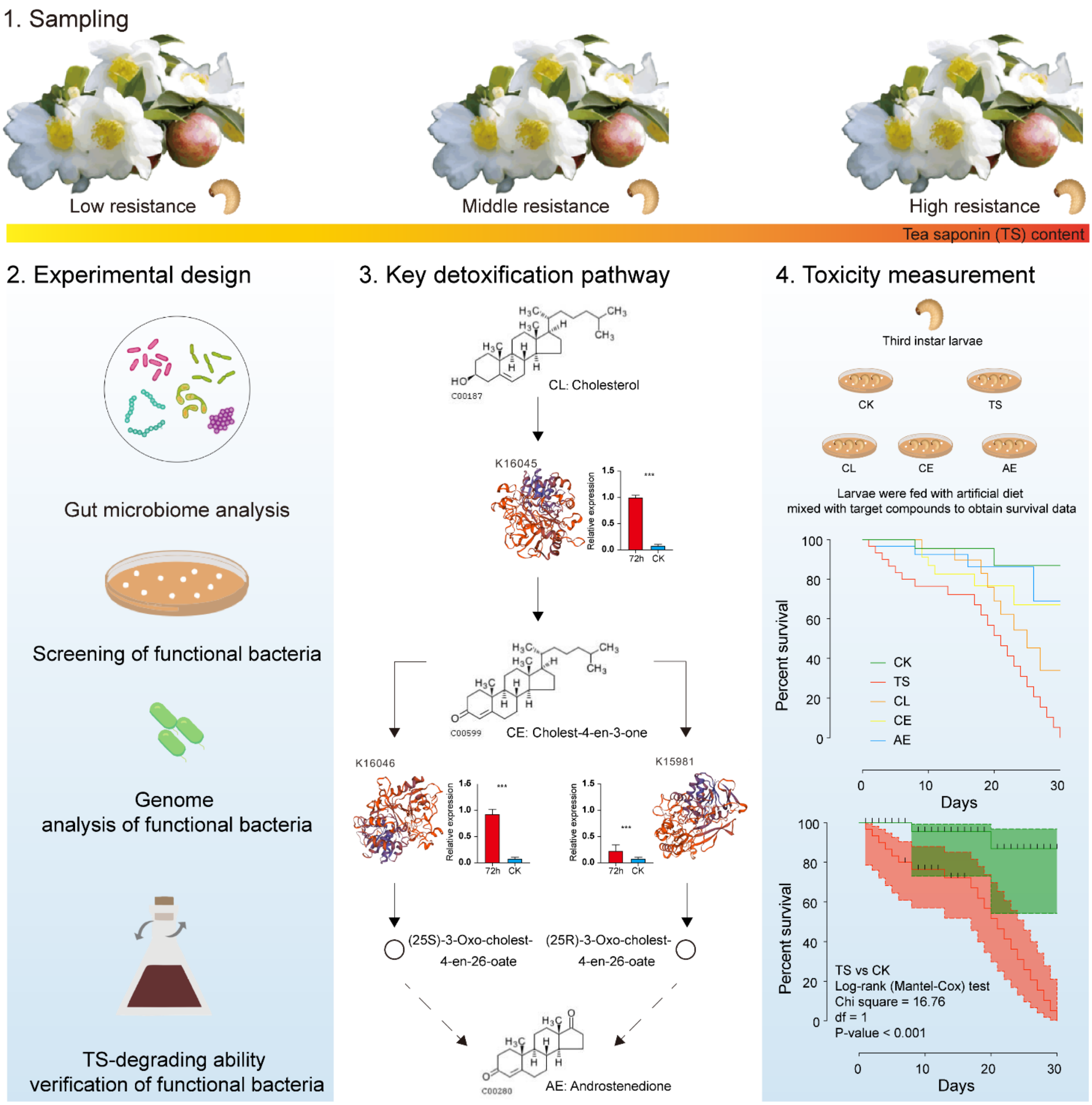

Fig. S2. Overview of sampling and design.

Table S1. Analysis of differences of alpha diversity index between groups

Groups	Shannon	Simpson	ACE	Chao1	PD_whole tree
High	3.678 ± 0.023a	0.860 ± 0.003a	64.273 ± 1.192a	64.167 ± 1.167a	2.997 ± 0.104a
Middle	2.796 ± 0.055b	0.782 ± 0.006b	67.136 ±1.791a	67.083 ± 1.772a	3.382 ± 0.109b
Low	2.474 ± 0.025c	0.735 ± 0.003c	57.905 ±1.949b	57.833 ± 1.887b	2.681 ± 0.058c

Table S2. Analysis of differences secondary metabolites between groups

Groups	Saponin mg/g	Tannic mg/g	Total hydroxybenzene mg/g	Organic acid mg/g
High-01	12.9	2.1	0.31	1.12
High-02	11.5	1.5	0.29	1.2
High-03	12.6	2.7	0.37	0.93
High-04	11.7	2.2	0.22	0.95
High-05	11.5	1.9	0.31	0.96
High-06	12.8	1.7	0.36	1.21
Middle-01	8.9	1.1	0.21	1.2
Middle-02	8.1	1.6	0.28	1.11
Middle-03	7.5	1.6	0.35	1.09
Middle-04	9.1	1.5	0.22	1.18
Middle-05	6.9	2.2	0.19	0.98
Middle-06	7.7	1.2	0.33	0.99
Low-01	2.9	1.9	0.31	0.91
Low-02	2.7	1.8	0.37	0.99
Low-03	3.5	2.2	0.22	1.06
Low-04	4.5	2.7	0.29	1.11
Low-05	3.2	2.1	0.27	1.18
Low-06	3.1	2.4	0.25	0.94

Table S3. Antibiotic resistance genes and Metabolism of terpenoids and polyketides genes used for comparative genomic analysis

Orthologous genes			Antibiotic resistance			Metabolism
A. baumannii	*A. lactucae*	AS23	Antibiotic resistance	Antibiotic target	Antibiotic biosynthesis	Metabolism of terpenoids and polyketides
A4U85_RS00230	HLV59_RS00230	gene41				+
A4U85_RS01580	HLV59_RS01635	gene316				+
A4U85_RS01895	HLV59_RS01960	gene386				+
A4U85_RS02415	HLV59_RS02480	gene486				+
A4U85_RS02420	HLV59_RS02485	gene487				+
A4U85_RS02425	HLV59_RS02490	gene488				+
A4U85_RS02430	HLV59_RS02495	gene489				+
A4U85_RS02435	HLV59_RS02500	gene490				+
A4U85_RS02955	HLV59_RS03025	gene595				+
A4U85_RS02960	HLV59_RS03030	gene596				+
A4U85_RS03005	HLV59_RS03075	gene604				+

A4U85_RS03415	HLV59_RS03520	gene693	+
A4U85_RS03560	HLV59_RS03665	gene724	+
A4U85_RS06435	HLV59_RS06385	gene1201	+
A4U85_RS06870	HLV59_RS06915	gene1301	+
A4U85_RS07150	HLV59_RS07245	gene1366	+
A4U85_RS07420	HLV59_RS07590	gene1437	+
A4U85_RS07465	HLV59_RS07640	gene1445	+
A4U85_RS07910	HLV59_RS08080	gene1538	+
A4U85_RS07925	HLV59_RS08095	gene1541	+
A4U85_RS07925	HLV59_RS08095	gene1580	+
A4U85_RS07965	HLV59_RS08135	gene1549	+
A4U85_RS08120	HLV59_RS08290	gene1541	+
A4U85_RS08120	HLV59_RS08290	gene1580	+
N/A	N/A	gene1752	+
A4U85_RS08800	HLV59_RS09455	gene1838	+

N/A	N/A	gene1839	+
N/A	N/A	gene1840	+
A4U85_RS08805	HLV59_RS09460	gene1841	+
N/A	N/A	gene1846	+
A4U85_RS08940	HLV59_RS09600	gene1883	+
A4U85_RS09895	HLV59_RS10500	gene2066	+
A4U85_RS10750	HLV59_RS11315	gene2232	+
A4U85_RS10810	HLV59_RS11375	gene2244	+
A4U85_RS10825	HLV59_RS11390	gene2247	+
N/A	N/A	gene2400	+
A4U85_RS11075	HLV59_RS11715	gene2441	+
A4U85_RS11090	HLV59_RS11730	gene2444	+
A4U85_RS14010	HLV59_RS13785	gene2816	+
A4U85_RS14015	HLV59_RS13790	gene2817	+
A4U85_RS14160	HLV59_RS13930	gene2845	+

A4U85_RS14865	HLV59_RS14510	gene2964			+
A4U85_RS16185	HLV59_RS15965	gene3237			+
A4U85_RS16750	HLV59_RS16485	gene3338			+
A4U85_RS17210	HLV59_RS16960	gene3433			+
A4U85_RS17215	HLV59_RS16965	gene3434			+
A4U85_RS18120	HLV59_RS17880	gene3609			+
A4U85_RS00020	HLV59_RS00020	gene4	+	+	
A4U85_RS00605	HLV59_RS00615	gene119	+	+	
A4U85_RS00730	HLV59_RS00745	gene143		+	
A4U85_RS00895	HLV59_RS00910	gene174		+	
A4U85_RS01225	HLV59_RS01250	gene244		+	
A4U85_RS02160	HLV59_RS02235	gene440	+		
A4U85_RS02815	HLV59_RS02875	gene566	+		
A4U85_RS03040	HLV59_RS03115	gene612		+	
A4U85_RS03380	HLV59_RS03480	gene679	+		
A4U85_RS03535	HLV59_RS03640	gene719	+		
A4U85_RS03540	HLV59_RS03645	gene720	+		
A4U85_RS03545	HLV59_RS03650	gene721	+		
A4U85_RS03570	HLV59_RS03670	gene725		+	
A4U85_RS03770	HLV59_RS03900	gene771	+		

A4U85_RS03775	HLV59_RS03905	gene772		+		
A4U85_RS03820	HLV59_RS03950	gene780	+			
A4U85_RS03930	HLV59_RS04060	gene798	+			
A4U85_RS03970	HLV59_RS04095	gene805		+		
N/A	HLV59_RS04180	gene815	+			
N/A	HLV59_RS04185	gene816	+			
A4U85_RS04125	HLV59_RS04290	gene840	+	+		
A4U85_RS04465	HLV59_RS04620	gene910		+		
A4U85_RS04835	HLV59_RS04975	gene980			+	
A4U85_RS05150	HLV59_RS05410	gene1043	+			
A4U85_RS05815	HLV59_RS05835	gene1100	+			
A4U85_RS05820	HLV59_RS05840	gene1101	+			
A4U85_RS05825	HLV59_RS05845	gene1102	+			
A4U85_RS05855	HLV59_RS05875	gene1108	+			
A4U85_RS06555	HLV59_RS06595	gene1239		+		
A4U85_RS07435	HLV59_RS07605	gene1439	+			
A4U85_RS07475	HLV59_RS07650	gene1447		+		+
A4U85_RS08660	HLV59_RS08405	gene1759	+			
A4U85_RS08665	HLV59_RS08400	gene1760	+			
A4U85_RS08670	HLV59_RS08395	gene1761	+			

A4U85_RS08675	HLV59_RS08390	gene1762	+		
A4U85_RS09145	HLV59_RS09860	gene1931		+	
A4U85_RS09920	HLV59_RS10525	gene2071	+		
A4U85_RS10615	HLV59_RS11175	gene2205		+	
A4U85_RS11195	HLV59_RS11780	gene2289	+		
A4U85_RS12085	HLV59_RS12645	gene2523	+		
A4U85_RS12275	HLV59_RS12880	gene2567	+		
A4U85_RS13835	HLV59_RS13595	gene2780	+		
A4U85_RS14050	HLV59_RS13820	gene2823	+		
A4U85_RS14080	HLV59_RS13850	gene2828			+
A4U85_RS14550	HLV59_RS14205	gene2904		+	
A4U85_RS14700	HLV59_RS14350	gene2934	+		
A4U85_RS14705	HLV59_RS14355	gene2935		+	
A4U85_RS14715	HLV59_RS14365	gene2937	+		
A4U85_RS16080	HLV59_RS15775	gene3198			+
A4U85_RS16115	HLV59_RS15800	gene3204	+		
A4U85_RS16445	HLV59_RS16185	gene3279	+		
A4U85_RS16700	HLV59_RS16440	gene3329	+		
A4U85_RS16785	HLV59_RS16520	gene3343	+		
A4U85_RS17295	HLV59_RS17050	gene3451	+	+	

A4U85_RS17300	HLV59_RS17055	gene3452	+	+
A4U85_RS17340	HLV59_RS17095	gene3459	+	
A4U85_RS17685	HLV59_RS17450	gene3525	+	+
A4U85_RS17715	HLV59_RS17485	gene3531	+	
A4U85_RS18165	HLV59_RS17945	gene3618		+
